# Supplementary material for: Mapping the Gut Microbiota Composition in the Context of Raltegravir, Dolutegravir, and Bictegravir—A Scoping Review
Source: Int J Mol Sci. 2025 Jul 2;26(13):6366. doi: 10.3390/ijms26136366 (PMC12250284; doi:10.3390/ijms26136366)
Supplement: Supplementary file 1 [file ijms-26-06366-s001.zip › Supplementary material 1.pdf]

**PubMed 2025.04.21.**

| Search | Query | Results |
|---|---|---|
| #3 | Search: **#1 AND #2** | 3,801 |
| #2 | Search: **("Gastrointestinal Microbiome"[Mesh]) OR (microbiome) OR (gut microbiome) OR (gut dysbiosis) OR (metagenomics) OR ("Metagenomics"[Mesh]) OR (microbiota)** | 220,253 |
| #1 | Search: **("HIV"[Mesh]) OR ("Acquired Immunodeficiency Syndrome"[Mesh]) OR (human immunodeficiency virus) OR (HIV) OR (opportunistic infections) OR (PLHIV) OR ("AIDS-Related Opportunistic Infections"[Mesh]) OR (AIDS) OR (acquired immunodeficiency syndrome) OR (bictegravir) OR (dolutegravir) OR (raltegravir) OR ("Raltegravir Potassium" [Mesh]) OR (tenofovir) OR ("Tenofovir" [Mesh]) OR (tenofovir alafenamide) OR (tenofovir disoproxil fumarate) OR (emtricitabine) OR ("Emtricitabine" [Mesh]) OR (lamivudine) OR ("Lamivudine" [Mesh]) OR (BIC) OR (DTG) OR (TAF) OR (TDF) OR (FTC) OR (3TC) OR (2DR) OR (INSTI) OR (integrase strand transfer inhibitor) OR ("HIV Integrase Inhibitors"[Mesh])** | 634,527 |

**Web of science 2025.04.21.**

| Search | Query | Results |
|---|---|---|
| #1 | Search: **(ALL=(microbiome) OR ALL=(gut microbiome) OR ALL=(gut dysbiosis) OR ALL=(metagenomics)) AND (ALL=(opportunistic infections) OR ALL=(PLHIV) OR ALL=(HIV) OR ALL=(human immunodeficiency virus) OR ALL=(AIDS) OR ALL=(acquired immunodeficiency syndrome) OR ALL=(bictegravir) OR ALL=(dolutegravir) OR ALL=(raltegravir) OR ALL=(tenofovir) OR ALL=(tenofovir alafenamide) OR ALL=(tenofovir disoproxil fumarate) OR ALL=(emtricitabine) OR ALL=(lamivudine) OR ALL=(BIC) OR ALL=(DTG) OR ALL=(TAF) OR ALL=(TDF) OR ALL=(FTC) OR ALL=(3TC) OR ALL=(2DR) OR ALL=(INSTI) OR ALL=(integrase strand transfer inhibitor))** | 4,769 |

**Embase 2025.04.21.**

| Search | Query | Results |
|---|---|---|
| #1 | Search: **((microbiome) OR ('gut microbiome') OR ('gut dysbiosis') OR (metagenomics)) AND ((HIV) OR (PLHIV) OR ('opportunistic infection') OR ('human immunodeficiency virus') OR (AIDS) OR ('acquired immunodeficiency syndrome') OR (bictegravir) OR (dolutegravir) OR (raltegravir) OR (tenofovir) OR ("tenofovir alafenamide") OR ("tenofovir disoproxil fumarate") OR (emtricitabine) OR (lamivudine) OR (BIC) OR (DTG) OR (TAF) OR (TDF) OR (FTC) OR (3TC) OR (2DR) OR (INSTI) OR ("integrase strand transfer inhibitor"))** | 4,569 |
